# Pulmonary Embolism and Increased Levels of D-Dimer in Patients with Coronavirus Disease

**DOI:** 10.3201/eid2608.201477

**Published:** 2020-08

**Authors:** Daniel O. Griffin, Alexandra Jensen, Mushmoom Khan, Jessica Chin, Kelly Chin, Jennifer Saad, Ryan Parnell, Christopher Awwad, Darshan Patel

**Affiliations:** Columbia University Medical Center, New York, New York, USA (D.O. Griffin);; Columbia University College of Physicians and Surgeons, New York (D.O. Griffin);; Donald and Barbara Zucker School of Medicine at Hofstra/Northwell, Hempstead, New York, USA (A. Jensen, M. Khan, J. Chin, K. Chin, J. Saad, R. Parnell, C. Awwad, D. Patel)

**Keywords:** coronavirus disease, COVID-19, severe acute respiratory syndrome coronavirus 2, SARS-CoV-2, viruses, respiratory infections, hypercoagulability, pulmonary embolism, thromboembolic, sequelae, d-dimer, deep vein thrombosis, prophylaxis

## Abstract

We report 3 patients with coronavirus disease who had a decline in respiratory status during their hospital course that responded well to intravenous steroids and interleukin-6 receptor antagonist therapy. These patients later showed development of persistent hypoxia with increased levels of d-dimer levels and were given a diagnosis of pulmonary embolisms.

Coronavirus disease (COVID-19), caused by severe acute respiratory syndrome coronavirus 2, has been extensively reported since the outbreak in Wuhan, China, and can progress to involve major respiratory complications (*1*). Patients commonly have fever, cough, abdominal pain, and diarrhea.

During the second week of illness, decompensation occurs in some patients, possibly driven by the cytokine storm associated with increased levels of interleukin-6. We report 3 case-patients with COVID-19 who were improving after successful treatment during the critical period but showed development of pulmonary emboli (PEs) despite deep vein thrombosis (DVT) prophylaxis.

Three patients admitted to Northwell Plainview Hospital (Plainview, NY, USA) showed positive results for COVID-19 and had acute hypoxic respiratory failure secondary to COVID-19. All 3 patients received azithromycin and hydroxychloroquine, but their conditions continued to progress to more severe respiratory failure. During what was assumed to be the cytokine storm phase, on the basis of laboratory parameters and an increasing requirement for oxygen, the patients received intravenous steroids (solumedrol, 1–2 mg/kg/d for 5–8 d) and the interleukin-6 receptor antagonist tocilizumab (400 mg intravenously). Patients showed improvement and did not require intubation but later showed development of persistent hypoxemia with increases in levels of d-dimer. Computed tomography angiograms (CTAs) confirmed bilateral PEs, and the patients required supplemental oxygen (Table).

**Table:** Characteristics of pulmonary embolism seen by CTA and increased levels of d-dimer in 3 patients with COVID-19, New York, USA*

Characteristic	Case-patient		
	1	2	3
Age, y	52	60	68
Risk factors	Allergic rhinitis, asthma	Chronic bronchitis, history of ovarian cancer, and history of provoked DVT	Hypertension, diabetes mellitus type 2
Smoking status	Former	Never	Never
BMI, kg/m^2^	27.0	27.4	23.7
Creatinine clearance, mL/min	116	127.4	64
Day of symptoms, baseline/CTA	12/18	8/18	14/22
O_2_ saturation, baseline/CTA	52% on RA/98% on NRB	92% on NC/91% on NC	94% on NRB/93% on NRB
d-dimer, μg/mL, baseline/CTA	2,283/9,698	221/2,563	33,318/1,554
Ferritin, μg/L, baseline/CTA	2,283/1,050	1,276/1,176	2,797/1,282
CRP, mg/L, baseline/CTA	32.30/0.42	11.89/0.66	8.88/0.25
Procalcitonin, ng/mL, baseline/CTA	0.19/0.05	0.05/0.13	0.23/NA
LDH, U/L, baseline/CTA	567/467	448/637	824/616
Neutrophil:lymphocyte ratio, baseline/CTA	10.58/11.75	6.6/7.5	7.67/14.99
ISTH score, day of CTA	>5	>5	>5
VTE prevention	Enoxaparin, 40 mg 2×/d	Enoxaparin, 40 mg 2×/d	Enoxaparin, 40 mg/d
IMPROV score	0	3	1
Doses of tocilizumab	1	1	1
Methylprednisolone duration, d	8	5	5
Hydroxychloroquine duration, d	5	5	5
CTA read	Bilateral PE; filling defects most pronounced in the right lobar pulmonary artery extending to the first-order branches of the right lower lobe pulmonary artery; additional small filling defect identified within the right upper lobe, right middle lobe, and lingular pulmonary artery branches; diffuse scattered bilateral ground-glass opacities with areas of consolidation compatible with reported viral pneumonia COVID-19	Multiple bilateral segmental and subsegmental PE with suggestion of cardiac strain; bilateral scattered, predominantly peripheral ground-glass opacities with some interlobular septal thickening consistent with given history of COVID-19 pneumonia	Central filling defects compatible with acute pulmonary embolism in several segmental and subsegmental pulmonary arteries in the right upper lobe, right lower lobe, and left lower lobe; diffuse bilateral ground-glass opacities unchanged from previous imaging

*BMI, body mass index; COVID-19, coronavirus disease; CRP, C-reactive protein; CTA, computed tomography angiogram; DVT, deep vein thrombosis; IMPROV, International Medical Prevention on Venous Thrombosis; ISTH, International Society of Thrombosis and Haemostasis; LDH, lactate dehydrogenase; NA, not available; NC, nasal cannula; NRB, nonrebreather; PE, pulmonary embolus; RA, room air; RLL, right lower lobe; VTE, venous thromboembolism.

Case-patient 1, a 52-year-old male former smoker with a history of asthma, came to our hospital 12 days after symptom onset. At admission, he reported chest tightness, difficulty breathing, and was afebrile. His respiratory rate was 34 breaths/min, heart rate 87 beats/min, and blood pressure 117/67 mm Hg. The d-dimer level was 2,283 μg/mL at admission and increased to 9,698 μg/mL on hospital day 6. He had been receiving enoxaparin (40 mg/d subcutaneously) as venous thromboembolism (VT) prophylaxis. He had worsening hypotension, dyspnea on exertion, chest discomfort, and shortness of breath. CTA performed on symptom day 18 showed bilateral PEs. The patient was given enoxaparin (1 mg/kg subcutaneously 2×/d), transitioned to rivaroxaban, and discharged receiving supplemental oxygen.

Case-patient 2, a 60-year-old female nonsmoker with a history of chronic bronchitis, ovarian cancer postoophorectomy, and provoked DVT 18 years earlier, was admitted on day 8 of symptoms. At admission, she reported worsening cough, nausea, and loss of sense of smell. She was afebrile; her respiratory rate was 20 breaths/min, heart rate 106 beats/min, and blood pressure 145/68 mm Hg. The d-dimer level was 221 μg/mL at admission and 2,563 μg/mL on hospital day 10. She was given DVT prophylaxis (enoxaparin, 40 mg/d subcutaneously, increased to 2×/d on day 10 of illness). On day 18 of symptoms, she was persistently hypotoxic and had tachycardia and hypotension. CTA showed multiple bilateral segmental and subsegmental PEs with suggestion of cardiac strain. The patient was given rivaroxaban and discharged receiving supplemental oxygen.

Case-patient 3, a 68-year-old male nonsmoker with a history of hypertension, and type 2 diabetes mellitus, was admitted on day 14 of symptoms. At admission, he reported cough, difficulty breathing, and progressive weakness. He was afebrile; his respiratory rate was 22 breaths/min, heart rate 107 beats/min, and blood pressure 144/92 mm/Hg, and he showed hypotoxicity. The d-dimer level was 33,318 μg/mL at admission and 1,554 μg/mL on hospital day-7. He was given DVT prophylaxis (enoxaparin, 40 mg/d subcutaneously). On day 22 of symptoms, he showed development of hypotension, and his oxygen saturation was <90% with a 100% nonrebreather mask. This finding prompted a CTA, which showed bilateral PEs on hospital day 3. The patient was given enoxaparin (1 mg/kg subcutaneously 2×/d) and showed improvement.

PEs can occur after the cytokine storm in COVID-19 patients, despite DVT prophylaxis. After initial improvements, patients might continue to have high or increasing oxygen requirements because of development of thromboembolic disease. Previous studies showed that low levels of platelets, increased levels of d-dimer, and increasing levels of prothrombin in COVID-19 were associated with poor outcome, which might be explained by thromboembolic complications in patients with severe disease (*2*). Platelet counts remained within reference ranges for 2 of our patients and only decreased for 1 patient. Two patients had increases in d-dimer levels, and the third patient was admitted with a highly increased d-dimer level.

Autopsy reports from COVID-19 patients have shown microthrombi in lungs and in other organs with associated foci of hemorrhage (*3*,*4*). These findings suggest that severe endothelial dysfunction, driven by the cytokine storm and associated hypoxemia, leads to disseminated intravascular coagulation, causing thromboembolic complications. In these patients, other parameters, such as the neutrophil–lymphocyte ratio, and inflammatory markers, including ferritin, C-reactive protein, and lactate dehydrogenase, were returning to reference levels despite increased d-dimer levels and increasing oxygen requirements. Standard dose DVT prophylaxis did not prevent this complication. This hypercoagulability was a consumptive coagulopathy and was not caused by an inhibitor, such as an anticardiolipin antibody; treatment with direct factor Xa inhibitors would be appropriate.

Although certain underlying conditions might have influenced the coagulation process in these patients, the hypothesis that hypercoagulability is driven by endothelial dysfunction is plausible. These case studies support the earlier observation that anticoagulation is associated with a decrease in mortality rates for COVID-19 patients (*5*). Monitoring disseminated intravascular coagulation and measurement of platelet counts, d-dimer and fibrinogen levels, and trending International Society of Thrombosis and Haemostasis scores might be beneficial for early diagnosis of PE in patients with COVID-19.
